# Systemically Administered Plant Recombinant Holo-Intrinsic Factor Targets the Liver and is not Affected by Endogenous B12 levels

**DOI:** 10.1038/s41598-019-48555-w

**Published:** 2019-08-22

**Authors:** Jayme L. Workinger, Akhila N. W. Kuda-Wedagedara, Mara M. Julin, Jordan M. White, Ebba Nexo, Nerissa T. Viola, Robert P. Doyle

**Affiliations:** 1 Department of Chemistry, 111 College Place Syracuse University, Syracuse, NY 13244 USA; 2 Department of Oncology, Karmanos Cancer Institute, Wayne State University, Detroit, MI 48201 USA; 3 Department of Clinical Biochemistry and Clinical Medicine, University of Aarhus, Aarhus, Denmark; 4 Department of Medicine, State University of New York (SUNY) Upstate Medical University, Syracuse, NY 13210 USA

**Keywords:** Diagnostics, Drug discovery and development

## Abstract

Precision targeting imaging agents and/or treatment agents to select cells or organs in the body remains a significant need and is an area of intense research. It has been hypothesized that the vitamin B12 (B12) dietary pathway, or components thereof, may be exploitable in this area. The question of whether gastric Intrinsic factor (IF), critical for B12 absorption in the GI tract via the cubilin receptor, could be used as a targeting moiety for the cubilin receptor *systemically*, has not been investigated. Cubilin is the only known receptor for holo-IF and is found primarily in the kidney and ear (outside of the ileum of the GI) offering significant scope for specific targeting. We utilized plant derived human gastric IF in fluorescent cell and PET based *in vivo* imaging and biodistribution studies and demonstrated that plant derived IF primarily targets the liver, likely a consequence of the unique glycosylation profile of the IF, and is not affected by endogenous B12 levels.

## Introduction

Utilization and exploration of vitamin B12 (B12) and its dietary pathway in pharmaceutical design and development has undergone a recent renaissance^1–8^. Several groups have now successfully used the pathway, or components thereof, to selectively target the peripheral nervous system over the central nervous system^3^, facilitate anti-platelet CO release^4^, target RNA to bacterial cells^5^, and image/treat cancer lines^6–8^. Development of B12 ‘antivitamins’ has also allowed for the greater exploration of the pathophysiological mysteries that still undermine our understanding of the effects of B12 deficiency^9,10^. Many critical areas necessary to open up the field to drug development have now also been well explored, including sites of B12 conjugation^11^, access to recombinant B12 transport proteins^12–14^ and conjugate binding affinities to such^15^, protective effects of B12 binding proteins on bound peptide conjugates^16^, temporal biodistribution across tissues *in vivo* in various B12 forms^17^, a clearer understanding of the types and locations of B12 binding proteins in animal models^18^, and the types and distribution of B12 cellular uptake receptors^19^. Other questions do remain, such as, whether utilizing the B12 dietary pathway over a prolonged period for drug delivery may be detrimental to B12 uptake in proliferating cells. Utilizing IF pre-bound B12 conjugates, however, would theoretically overcome this scenario, since IF does not play a role systemically in B12 cellular uptake.

Critically, a basic understanding of the dietary pathway is also in place^20^. Transport and delivery of B12 utilizes three primary carrier proteins: haptocorrin (HC; K_d_ = 0.01 pM), intrinsic factor (IF; K_d_ = 1 pM), and transcobalamin (TC; K_d_ = 0.005 pM), each responsible for carrying a single B12 molecule^20^. IF is a ~50 kDa glycosylated protein that is secreted from parietal cells of the gastric mucosa and is resistant to pancreatic enzymes^16,20^.

Once B12 is bound to IF, it facilitates intestinal transport and passage across the ileal enterocyte. This passage occurs via receptor-mediated endocytosis through the IF-B12 receptor cubilin (CUBN) combined with a transmembrane protein, amnionless (CUBAM)^21,22^. Following internalization, IF is degraded by lysosomal proteases and B12 is released into the blood stream, either as free B12 or pre-bound to TC^20,23^. Cells that require B12 express the holo-TC receptor, CD320. Upon internalization, TC is degraded and B12 is transported from the lysosome for cellular use.

Herein, we sought to investigate the effects of *systemic* administration of B12 conjugates *pre-bound* to recombinant human gastric IF. The first outcome postulated would be that IF pre-binding would facilitate targeting the only known holo-IF receptor, CUBN, located in the ileum in the enterocyte, as described for dietary uptake, but also in the proximal tubules (PT) of the kidney, where it now partners with megalin and plays a role in reabsorption of such ligands as albumin, transferrin, vitamin D binding protein, apolipoprotein AI, amongst others^24^. We also postulated that IF pre-binding would *prevent* TC binding and hence would not be affected by endogenous B12 levels, a long-time concern in the field given binding to TC results in significant back-ground across tissues and offers the possibility of causing a loss of B12 cellular delivery (TC dependent)^25^. Expression of CUBN elsewhere is limited, including the human inner ear^26^ and yolk sac^27^.

Before beginning such work, it was necessary to ensure access to IF that (1) was available commercially on a large-scale (i.e. 30–50 mg quantities) necessary to conduct, and ultimately translate the work, and (2) that it was in the apo- (i.e. no pre-bound B12) form, to allow binding of the desired B12-conjugates, which in this case are radio-probes of ^89^Zirconium-B12 ([^89^Zr]-B12), *vide infra*^28^. To achieve this, the only available source meeting our criteria was human recombinant IF (IF) produced in the plant *Arabidopsis thaliana*^29^. Expression in plants produces apo-IF, given plants are a rare organism that do not use B12, minimizing holo-IF production *in situ*. Questions to be explored with *A*. *thaliana* produced IF included the glycosylation profile of such a protein and the effects of such glycosylation on receptor targeting *in vivo*, as noted above, and whether this profile negated, complemented or refocused the CUBN targeting hypothesis noted above.

## Results

B12-DFO and B12-DFO-[^89^Zr] ([^89^Zr]-B12**)** were synthesized and characterized as previously reported with a final yield of 20 and 100%, respectively^28^. The specific activity of the tracer for studies herein was determined by titrating [^89^Zr^4+^] and B12-DFO at different mole ratios with an achieved optimum specific activity of 250 ± 20 mCi/µmol. Stability of the tracer was analyzed by incubating the IF-^89^Zr-B12 in saline at physiological temperature and analyzing fractions up to 24 h using iTLC (Fig. S3). Results indicated that the IF-[^89^Zr]-B12 tracer was stable to demetallation up to 24 h.

**Figure 1:**
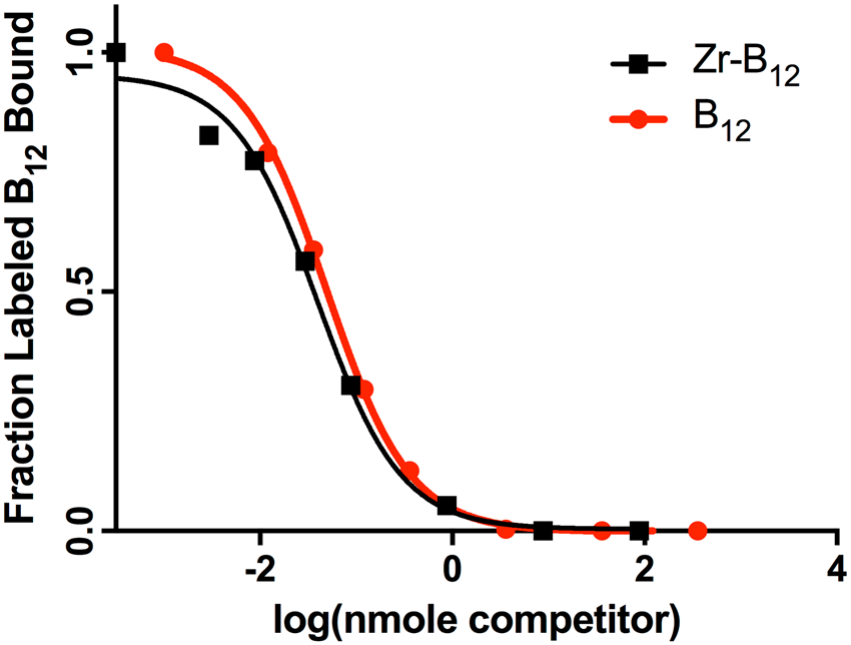
Binding affinities of [^91^Zr]-B12 and CN-B12 to human gastric IF with a K_d_ observed of 1.57 nM and 1.36 nM, respectively.

To confirm IF binding of [^89^Zr]-B12, a radiometric chase assay^15^, was completed with a ‘cold’ ^91^Zr bound to B12 tracer ([^91^Zr]-B12) and compared to free B12, as cyanocobalamin (CN-B12) (Fig. 1). [^91^Zr]-B12 was made using B12-DFO and chelated to [^91^Zr]Cl_4_ at pH 7–7.5. IF binding of [^91^Zr]-B12 was maintained at low nanomolar levels (1.57 nM), similar to CN-B12 control (1.36 nM).

The glycosylation of IF was examined by GC-MS (Table 1 and Supplementary Fig. S4). The sugars identified were α(1–3)-fucose, xylose, mannose and *n*-acetylglucosamine in the ratios 0.17: 0.18: 1.0: 0.24, respectively.

**Table 1:** GC-MS analysis of hrIF expressed in *A*. *thaliana*.

Sugar	Peak Area Ave.	[nmol] Detected	Ratio (Man = 1.0)
Fucose	8471	11.2	0.17
Xylose	6156	11.8	0.18
Mannose	81654	66.7	1.0
*n*-acetylglucosamine	4508	16.0	0.24

**Figure 2:**
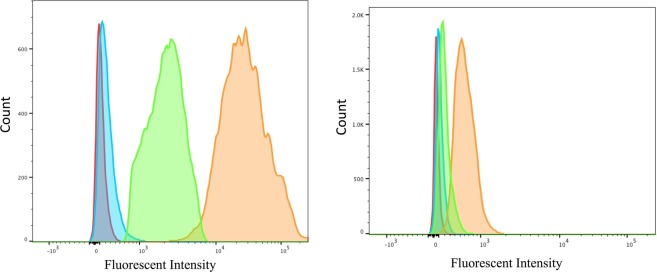
Flow cytometry analysis in (Left) in BN16 cells treated with IF-B12-Cy5 (orange), B12-Cy5 (blue) (200 nM each) at 37 °C and IF-B12-Cy5 (200 nM) at 4 °C (green) in HBSS for 1 h. Untreated cell background fluorescence is indicated in red; (Right) J774A.1 cells treated with IF-B12-Cy5 (orange), B12-Cy5 (blue) (200 nM each) and IF-B12-Cy5 cells with mannan block (green) (2 mg/mL) in HBSS for 1 h at 37 °C.

Cellular association via the typical holo-IF target receptor, CUBN, was conducted in CUBN positive, CD206 negative (see western blot, Fig. S5) BN16 (Brown Norway rat yolk) cells via flow cytometry using fluorescent B12-Cy5 to show functionalization of the IF-B12 complex *in vitro* (Fig. 2). Results showed no association of B12-Cy5 alone, and significant association of IF-B12-Cy5 at 37 °C. Reduction in binding (or internalization) of IF-B12-Cy5 at 4 °C supported a receptor mediated internalization. No association/binding was observed in Chinese hamster ovary (CHO) cells (CUBN and CD206 free cells; CD320 (TC receptor; XM_027442179.1)+; Figure S7) or in ASGPR positive (Figure S6) HepG2 cells (Figure S8).

Then, we investigated uptake in J774.A1 macrophage cells (CUBN- and CD206+)^30^, which again showed no binding of B12-Cy5 alone, and binding of IF-B12-Cy5 at 37 °C. Adding mannan (2 mg/mL), 45 minutes prior to, and concomitant with IF-B12-Cy5 incubation, reduced IF-B12-CY5 uptake (Fig. 2) supporting a mannose receptor mediated process.

**Figure 3:**
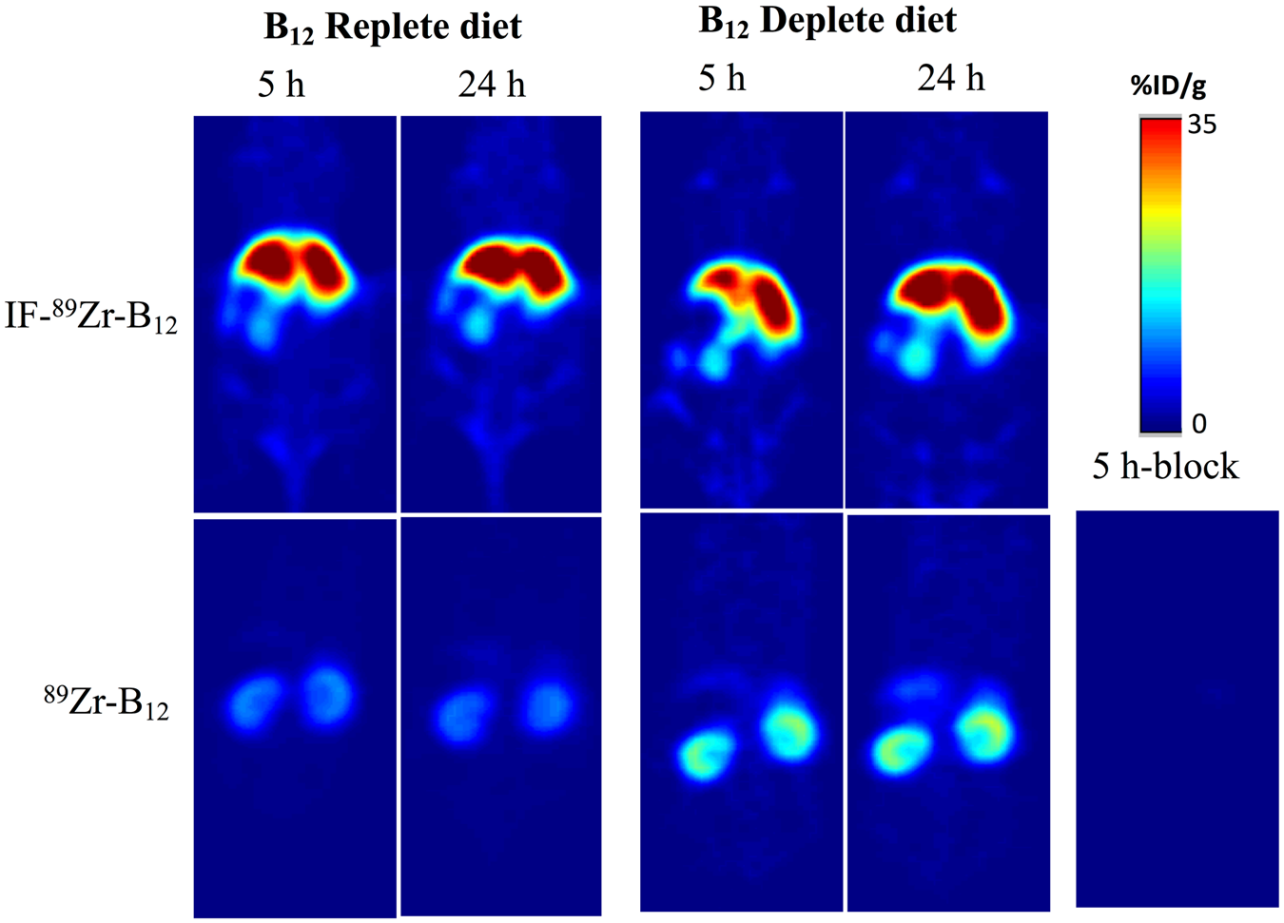
PET images of representative nude athymic mice on B12 replete (left) and deplete (right) diets after injections of [^89^Zr]-B12 or IF-[^89^Zr]-B12 at 5 and 24 h p.i plotted as %recovered/organ mean ± SD. Bottom right hand corner shows 5-h block using cold B12-DFO tracer in large excess (200 nM; 200-fold tracer concentration) in animals on replete diet.

Upon completion of the synthesis and characterization of the [^89^Zr] conjugate of interest, we began PET imaging studies. Initially, PET imaging was completed in nude athymic female mice on replete chow containing B12 at 1, 5 and 24 h p.i. of IF-[^89^Zr]-B12 (200–250 μCi/mouse via the tail vein, as for [^89^Zr]-B12). As shown in Fig. 3 and Table S1 there was significant liver uptake at 5 h, which did not change over the subsequent 24 h. Experiments were duplicated in mice on a B12 deplete diet for 21 days. For IF-[^89^Zr]-B12 the highest uptake was seen in the liver and kidneys and did not look significantly different than mice on replete diets (Fig. 3). However, in comparison to [^89^Zr]-B12 a change was observed with reduced kidney uptake noted in deplete animals (Fig. 3; Table S1).

**Figure 4:**
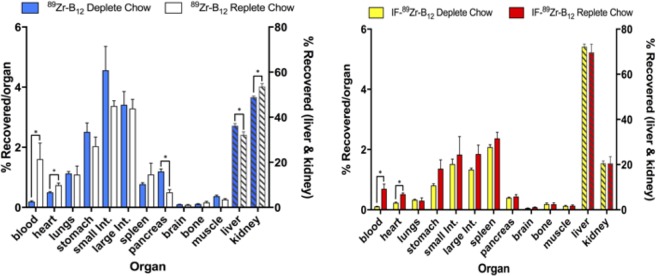
*Ex vivo* tissue distribution of [^89^Zr]-B12 (left) and IF-[^89^Zr]-B12 (right) in mice (n ≥ 3) on a B12 deplete or replete diet at 24 h plotted as %recovered/organ mean ± SD. [^89^Zr]-B12 showed significant changes occurred in liver, kidneys, blood, pancreas, and heart between the two mice models while IF-[^89^Zr]-B12 only showed significant changes occurred in blood, and heart.

**Figure 5:**
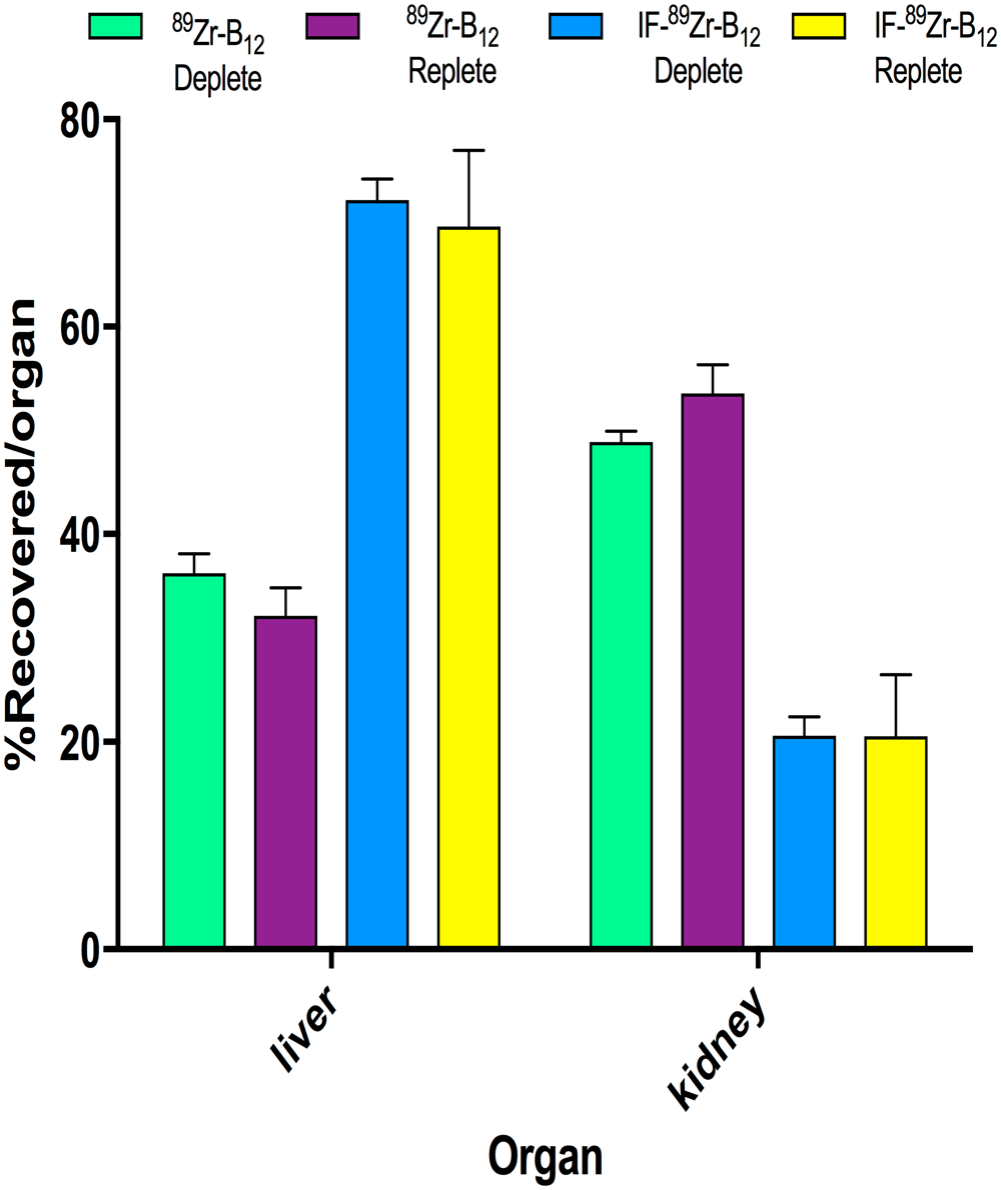
*Ex vivo* tissue distribution of IF-[^89^Zr]-B12 and [^89^Zr]-B12 in mice on a B12 deplete or replete diet at 24 h plotted as %recovered/organ as mean ± SD. The significant changes occurred with [^89^Zr]-B12 in the liver and kidney, while they were not significantly changed in the IF-[^89^Zr]-B12. n ≥ 3, *p ≤ 0.05.

Due to the interesting uptake seen in PET imaging using IF-[^89^Zr]-B12 in mice, *ex vivo* distribution was examined (Figs. 4 and 5 and Table S1). [^89^Zr]-B12 replete and deplete showed significant change in uptake within the liver, kidneys, blood, pancreas, and heart between the two mice models (B12 replete and deplete diets) (liver: 32.18 ± 2.6 vs 36.24 ± 1.8, kidney: 53.58 ± 2.7 vs 48.89 ± 1.0, blood: 1.60 ± 1.0 vs 0.192 ± 0.05, pancreas: 0.489 ± 0.18 vs 1.19 ± 0.15, heart: 0.740 ± 0.14 vs 0.501 ± 0.05, % recovered/organ for replete vs deplete; p ≤ 0.05, n = 4) (Table S1).

The IF-[^89^Zr]-B12 injected mouse models showed significant change in uptake within the blood, and heart (blood: 0.69 ± 0.31 vs 0.106 ± 0.01, heart: 0.51 ± 0.09 vs 0.23 ± 0.04% recovered/organ for replete vs deplete; p ≤ 0.05, n ≥ 3). IF-[^89^Zr]-B12 uptake in the liver, kidneys, spleen, and pancreas were not significantly different between the two models (liver uptake: 69.67 ± 7.3 vs 72.22 ± 2.0, kidneys: 20.56 ± 5.9 vs 20.61 ± 1.8, spleen: 2.37 ± 0.40 vs 2.07 ± 0.14, and pancreas: 0.43 ± 0.12 vs 0.399 ± 0.03% recovered/organ for replete vs deplete) (Table S1).

## Discussion

We first characterized the apo-IF’s glycosylation profile from *A*. *thaliana* given the postulated differences in glycol profile for human versus plant IF, and the role such sugars can play in terms of receptor recognition and binding, and protein clearance. GC-MS data showed a plant glycosylation profile of α(1–3)-fucose, xylose, mannose and *n*-acetylglucosamine in the ratios 0.17: 0.18: 1.0: 0.24, respectively. Since galactose was not detected the most likely receptor causing the liver internalization of IF was the mannose receptor CD206, which recognizes fucose, mannose, and *n*-acetylglucosamine and is found in liver epithelial cells and macrophages^31^. ASGPR is also highly expressed in the liver, however, this receptor recognizes terminal galactose, which was not present on the hrIF used herein^32^. Since this differs from a human glycosylation profile we wanted to investigate the hypothesis that the glycosylation profile might alter IF recognition in the body (should be only recognized by CUBN) and be recognized by the CD206.

To test our hypothesis, we first completed *in vitro* experiments with a fluorescent B12 conjugate, B12-Cy5, synthesized previously^3^, to allow the performance of quantitative flow cytometry experiments. We confirmed our IF-B12-Cy5 functioned as endogenous IF and that it was recognized by CUBN in the CUBN+ cell line BN16. Then, we investigated uptake in J774.A1 macrophage cells (CUBN- and CD206+), which indicated that IF-B12-Cy5 recognition is IF specific and supports the GC-MS sugar profile. A prior report by Paveley *et al*.^30^, supports the results, with a similar shift in fluorescence observed when a fluorescent-monoclonal antibody for the CD206 receptor was incubated with J774A.1 cells. In addition, a near complete block in uptake was observed when J774A.1 cells were incubated with an excess (2 mg/mL) of mannan, which is reported to reduce CD206 mediated uptake^33^, 45 minutes prior to incubation with IF-B12-Cy5, supporting that the uptake is mediated via the CD206 receptor (Fig. 2). Also important to note that macrophages expressing high levels of CD206 have been considered as anti-inflammatory and therefore targeting this receptor could be toxic with prolonged administrations *in vivo*^34^. CUBN and CD206 negative cell line CHO-K1 (confirmed by Western blot- data not shown) did not show any association (Fig. S7).

Since the *in vitro* studies confirmed our hypothesis, we continued our investigation *in vivo* using PET imaging. Upon completion of the synthesis, characterization, and stability studies of [^89^Zr]-B12 and IF-[^89^Zr]^_^B12 (Figs. S1–S3) indicated that *in vivo* PET imaging studies could be conducted. Initially, PET imaging was completed in nude athymic female mice on replete chow containing B12 at 1, 5, and 24 h p.i. (200–250 μCi/mouse via the tail vein) of IF-[^89^Zr]-B12 (data not shown). As shown in Fig. 3 and Table S1, there was significant liver uptake at 5 h, which did not change over the subsequent 24 h. Overall, the highest uptake was observed in the liver, compared to the control ([^89^Zr]-B12 alone) which showed uptake primarily in the kidneys.

To more closely examine the effects of B12 status on distribution, IF-[^89^Zr]-B12 or [^89^Zr]-B12 were injected into nude athymic female mice on a B12 deplete diet for 21 days and PET imaging was completed on mice 24 h p.i. For IF-[^89^Zr]-B12 the highest uptake was seen in the liver and kidneys and did not look significantly different than mice on replete diets. However, in comparison to [^89^Zr]-B12 a clear change was observed with higher uptake in the liver. This is in accord with the current view, that in rodents the kidneys store excess B12^24,35^. To quantify this change, biodistribution studies were conducted (Figs. 4 and 5 and Table S1).

[^89^Zr]-B12 replete and deplete biodistribution showed significant change in uptake within the liver, kidneys, blood, pancreas, and heart (p < 0.05). The IF-[^89^Zr]-B12 replete and deplete models showed significant change within the blood, and heart (p < 0.05). To date most B12 experiments show high uptake in the kidneys with less uptake in the liver, our data displays an altered pharmacokinetic (PK) and uptake profile for the IF-bound B12. This change in PK is most likely, in part, due to the CD206 receptor, highly expressed in the liver and macrophages^36,37^, which recognize the specific glycosylation profile of *A*. *thaliana* produced recombinant human IF.

So, while we did not set out to target the liver, this is what was ultimately observed. This observation has implications for drug delivery using plant derived IF, depending on how easily a bound B12-conjugate drug could be unloaded, and which cells took it up. Such work is on-going in our labs.

In conclusion, the absence of effect on IF uptake by endogenous B12 levels indicates that IF can allow for the use of B12 conjugate chemistry (i.e. B12 drug conjugates) while stepping out of the normal B12 ‘dietary’ pathway dependent on TC mediated cellular uptake. This use of IF would diminish any possible risk, however unlikely (and certainly not confirmed to date), of developing B12 deficiency in subjects being dosed with B12 bioconjugates. The liver uptake seen in PET imaging and biodistribution when a radio-B12 complex of IF was administered was attributed to the terminal sugar being recognized by, most likely, the CD206 receptor, itself a major target for pharmaceutical intervention/targeting.

## Methods

Reagents listed below were purchased and used without further manipulations: Dimethyl sulfoxide (DMSO, 99%, Sigma), Vitamin B_12_ (Cbl, ≥98%, Sigma), 1,10-carbonyl-di-(1,2,4-triazole) (CDT, ≥90%, Fluka), and acetonitrile (MeCN, 99.8%, Pharmaco-Aaper), desferrioxamine mesylate (Sigma), F12-K media (VWR), Dulbecco’s modified eagles medium (DMEM) (VWR), mannan (VWR). Western blotting: Samples were run on a 12% acrylamide gel and then transferred to a nitrocellulose membrane using an iBlot (Invitrogen) dry blotting system. The membrane was blocked in a 5% nonfat powdered milk PBS-T solution (w/v) for one hour at room temperature prior to western blotting. Antibodies: 1° Santa Cruz Biotechnology cubilin anti-goat polyclonal (1:200); Santa Cruz Biotechnology chicken anti-goat HRP conjugated (1:4000); anti-mannose (CD206) receptor antibody (abcam, ab64693); anti-asialoglycoprotein receptor (abcam, ab88042), HRP-conjugated goat anti-rabbit (abcam, ab6721). RP-HPLC was performed using either an Agilent 1200 system or a Shimadzu Prominence with an Agilent Eclipse C_18_ XBD analytical column (5 μm x 4.6 mm x 150 mm) using a 0–70% 0.1% aqueous TFA to MeCN gradient over 30 minutes. Proton nuclear magnetic resonance (^1^H NMR) was performed using a 400 MHz Bruker spectrometer with the residual non-deuterated solvent peak as an internal standard. Matrix assisted laser desorption ionization mass spectrometry (MALDI-MS) was conducted on a Bruker Autoflex III smartbeam using sinapinic acid (Sigma) as matrix. Quantification in solution used a Shimadzu BioSpec-Nano. Flow cytometry analyses were carried out on a Becton Dickinson LSRII Cell Analyzer. Cell lines J774A.1 (ATCC TIB-67; CD206 positive), CHO-K1 (ATCC CCL-61; control line) and HepG2 (SIGMA 85011430; ASGPR positive) were obtained from the American Type Culture Collection (ATCC). BN16 cells (cubilin positive) were kindly provided by Prof. Pierre Verroust (INSERM, Paris, France). J774A.1 and BN16 cells were cultured as adherent monolayers in DMEM supplemented with 10% FBS and 1% pen/strep (Penicillin-streptomycin solution with 10,000 units penicillin and 10 mg/mL streptomycin in 0.9% NaCl obtained from Thermo Fisher). CHO-K1 were cultured as adherent monolayers in F12-K supplemented with 10% FBS and 1% pen/strep. Cells were incubated at 37 °C with 5% CO_2_. Hank’s balanced salt solution (HBSS) was purchased from Sigma. Charcoal stripped fetal bovine serum (FBS) and were purchased from Sigma. Xeragenx LLC (St. Louis, MO, USA) supplied the apo-hrIF expressed in *A*. *thaliana*. Analysis of the radiotracer was performed using C18 reverse phase high-pressure liquid chromatography (RP-HPLC, Agilent 1260 with manual injection) and instant thin layer chromatography (iTLC, Eckert & Ziegler Mini Scan). EDTA (50 mM) mobile phase was used for iTLC. Female athymic nude mice (5–6 weeks old) were purchased from Envigo (Catalog# 069).

All animal experiments and manipulations were carried out after review by, and with the approval of, the IACUC committee at Wayne State University. All animal experiments and manipulations were carried out according to the guidelines and regulations set by the IACUC at Wayne State University, which is accredited by the Association for Assessment and Accreditation of Laboratory Animal Care (AAALAC). IACUC Protocol # for this work was 17-07-302.

### Synthesis of B12-DFO and B12-DFO-[^89^Zr]

**Figure 6:**
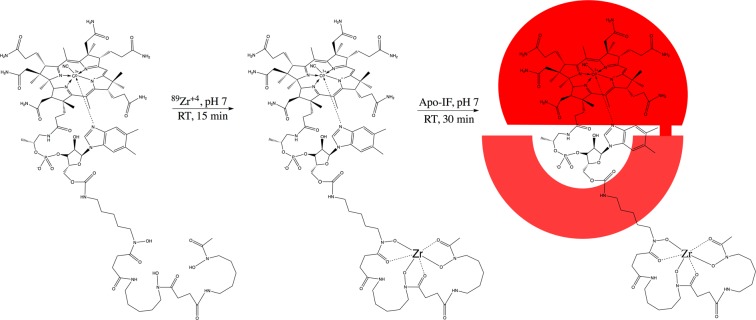
The B12-DFO conjugate was first incubated with ^89^Zr at neutral pH at room temperature for 15 min according to previously published protocols^28^. After confirmation of binding through iTLC, [^89^Zr]-B12-DFO was incubated with a slight excess of apo-IF (indicated in red) for 30 min then purified with a 30 kDa spin filter (GE Vivaspin).

B12-desferrioxamine (B12-DFO) and B12-DFO-[^89^Zr] ([^89^Zr]-B12**)** were synthesized and characterized as previously reported^28^. Optimum conditions for radio labeling of B12-DFO were tested by titrating with ^89^Zr and analyzing the incubated solution using iTLC. Approximately 1 mCi (37 MBq) of [^89^Zr](C_2_O_4_)_2_ (3D imaging, AZ) was diluted with 0.9% saline and the pH was adjusted to 7 by adding 1 M Na_2_CO_3_. A solution of B12-DFO (0.004 µmol, 10.8 µg) was added to the pH adjusted ^89^Zr acetate solution and incubated for 15 min at room temperature (RT) (Fig. 6). The identity of the tracer was characterized via MALDI-MS analysis using B12-DFO labeled with ‘cold’ ^91^Zr^4+^ (Fig. S1), as control; Expected: 2030.2 [M^+^]; observed: 2005.2 [M-CN + H]^+^.

### Binding [^89^Zr]-B12 to IF

A 1:0.8 ratio (apo-IF:[^89^Zr]-B12-DFO) was used for binding. The radiolabeled compound was incubated with IF for 30 min at neutral pH at room temperature, then purified through a 30 kDa size exclusion spin filter volume (GE Vivaspin), adjusted with saline solution. Radio labeling efficiency of >97% was determined by iTLC (Fig. S2). Stability was confirmed over 24 hours in saline (Fig. S3).

### Stability of IF-[^89^Zr]-B12

Stability of IF- [^89^Zr]-B12 was tested by incubating the tracer (200 µCi, 100 µl) in saline (0.9% NaCl) (Sigma) at 37 °C and fractions (50 µCi) were analyzed for free ^89^Zr at 1, 4, and 24 h intervals using radio-HPLC (Agilent) and iTLC.

### IF and TC binding affinities

To confirm that [^89^Zr]-B12 will bind to IF and TC (see Fig. S9 for source), a radiometric chase assay, using ^57^Co-B12 was completed (as previously reported)^15^ with a cold tracer ([^91^Zr]-B12) and compared to free B12, as cyanocobalamin (CN-B12). Zr-B12 was made using B12-DFO and chelated to ZrCl_4_ at pH 7.5.

### Synthesis of B12-Cy5

B12-Cy5 was synthesized and characterized as previously reported^3^. Yield: 94%.

### Flow cytometry measurements of cellular internalization

Cells were plated on a 6-well plate and allowed to adhere for at least 24 h until at least 80% confluency. Cells were washed 3x with HBSS and then incubated with 1 mL of IF-B12-Cy5, B12-Cy5 (200 nM) or HBSS without any conjugate unless otherwise indicated for 1 h and then washed in triplicate with HBSS. Cells were stripped mechanically and 1 mL of media was added and analysis performed. All cells were excited at 640 nm and detected at 660 ± 20 nm.

#### GC-MS Analyses of the Glycosylation profile of Recombinant Human IF Expressed in A. thaliana

Samples were analyzed by SGS M-Scan Inc. (West Chester, PA, USA) by GC/MS. Key table generated in report is included as Fig. S4.

### PET imaging experiments

[^89^Zr]-B12 was intravenously administered (200–250 µCi/mouse, 0.8–1 nmol) in sterile saline in female nude mice on a B12-deplete (Envigo (Teklad) custom B12-free diet) or B12-replete diet (regular chow) for 3 weeks. A µPET scanner (Siemens Concord) was used for PET imaging and was initially completed at 1, 4, and 24 post-injection (p.i.) time points while the mice were anesthetized with 1–2% isoflurane (Baxter, Deerfield, IL) however, due to the similarity of the scans and background clearance, only 24 h p.i was used throughout the rest of the experiments. Images were reconstructed using filtered back projection algorithm. ASIPro VMTM software version 6.3.3.0 (Concord) was used to analyze the images to acquire volumes-of-interest expressed as % injected dose per gram of tissue (%ID/g).

### ***Ex vivo*** distribution

The tissue distribution of [^89^Zr]-B12 was studied by administering 10–25 µCi (0.04–0.1 nmol) of the tracer on the lateral tail vain of the rodent. Euthanasia via CO_2_ asphyxiation was performed at 1, 4, and 24 h p.i.

## Supplementary information

Supplementary materials
