# Supplementary material for: Systemically Administered Plant Recombinant Holo-Intrinsic Factor Targets the Liver and is not Affected by Endogenous B12 levels
Source: Sci Rep. 2019 Aug 22;9:12269. doi: 10.1038/s41598-019-48555-w (PMC6706418; doi:10.1038/s41598-019-48555-w)
Supplement: Supplementary file 1 — Supplementary materials [file 41598_2019_48555_MOESM1_ESM.docx]

Systemically Administered plant recombinant holo-Intrinsic Factor Targets the Liver and is not Affected by Endogenous B12 levels

Jayme L. Workinger,^1^† Akhila N. W. Kuda-Wedagedara,^2^† Mara M. Julin,^1^ Jordan M. White,^2^ Ebba Nexo,^3^ Nerissa T. Viola^2^*, and Robert P. Doyle,*^1,4^

^1^ Department of Chemistry

111 College Place

Syracuse University, Syracuse, NY 13244 (USA)

^2^ Department of Oncology

Karmanos Cancer Institute,

Wayne State University, Detroit, MI 48202 (USA)

^3^Department of Clinical Biochemistry and Clinical Medicine,

University of Aarhus, Aarhus, Denmark

^4^ Department of Medicine

State University of New York (SUNY) Upstate Medical University

Syracuse, NY 13210 (USA)

Corresponding Authors^*^

Nerissa T. Viola and Robert P. Doyle

† Contributed equally to this work.

Running Title: *Systemically administered holo-Intrinsic Factor*

**SUPPORTING INFORMATION**

**Figure S1.** MALDI-MS analysis of B12-DFO bound to cold Zr^4+^. Expected: 2030.2 [M^+^]; observed: 2005.2 [M-CN+H]^+^.

**Figure S2.** iTLC of IF-^89^Zr-B12 solution after 30 min incubation with a 1:0.8 excess of IF to ^89^Zr-B12. Results indicate all ^89^Zr-B12 was bound by IF and no loss of ^89^Zr was observed.

**Figure S3.** iTLC of IF-^89^Zr-B12 stability at 0, 1, 4, and 24 h incubation with saline at 37°C. Results indicated complex was stable with no loss of tracer noted. Shift in peak was attributed to unaligned spotting.


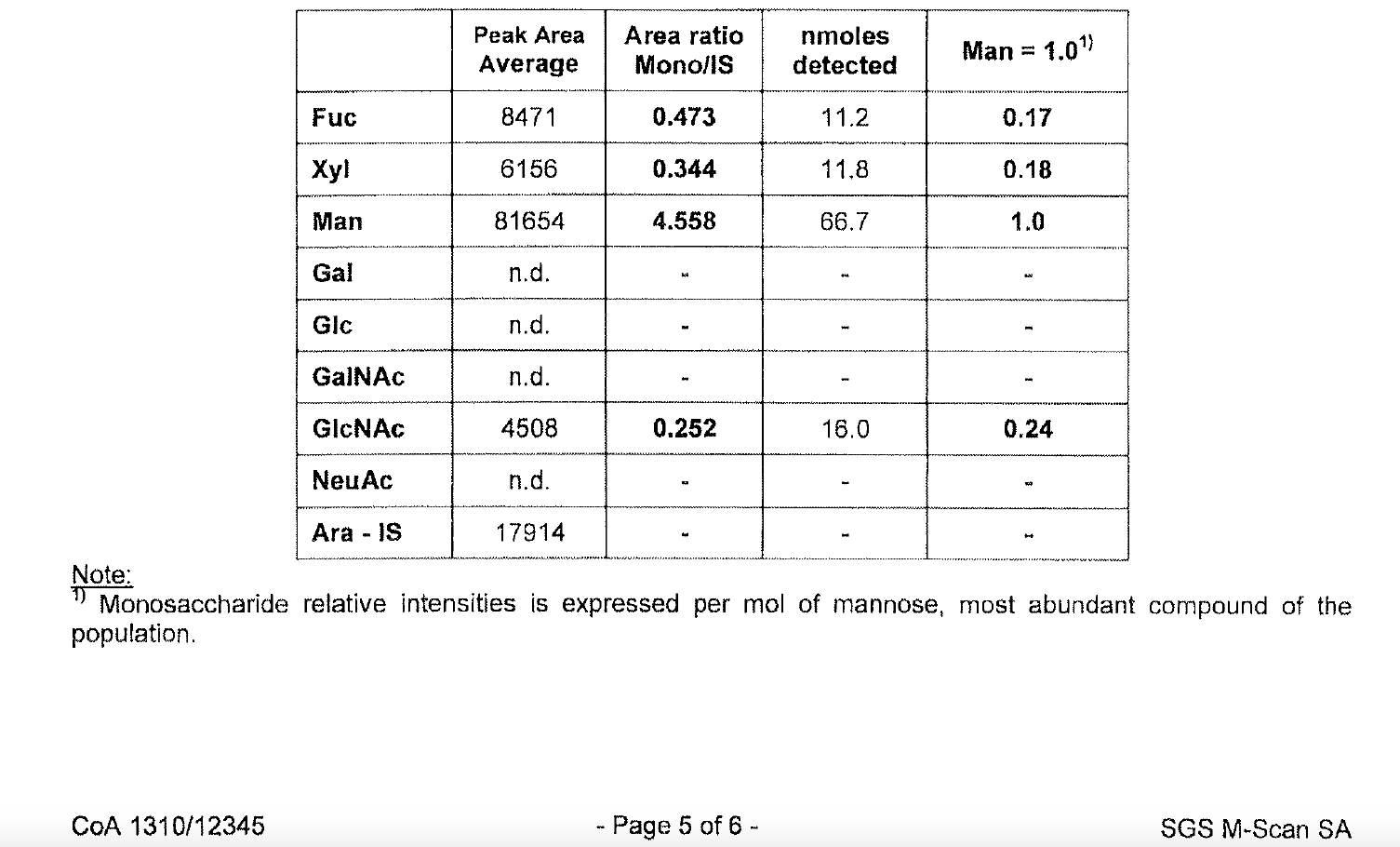


**Figure S4.** Results of GC-MS determined IF glycosylation profile. Only fucose, xylose, mannose, and n-acetylglucosamine were detected and at a ratio of 0.17, 0.18, 1.0, 0.24, respectively.

| Organs | ^89^Zr-B12  Replete | IF-^89^Zr-B12  Replete | ^89^Zr-B12  Deplete | IF-^89^Zr-B12  Deplete |
| --- | --- | --- | --- | --- |
| Blood | 1.60 ± 1.07**^a^** | 0.72 ± 0.26**^b^** | 0.19 ± 0.05**^a^** | 0.106 ± 0.01**^b^** |
| Heart | 0.74 ± 0.14**^a^** | 0.51 ± 0.09**^b^** | 0.50 ± 0.05**^a^** | 0.23 ± 0.04**^b^** |
| Lungs | 1.09 ± 0.57 | 0.29 ± 0.19 | 1.12 ± 0.12 | 0.32 ± 0.03 |
| Liver | 32.18 ± 2.61**^a^** | 69.67 ± 7.34 | 36.24 ± 1.88**^a^** | 72.22 ± 2.02 |
| Kidney | 53.58 ± 2.72**^a^** | 20.56 ± 5.90 | 48.88 ± 1.01**^a^** | 20.61 ± 1.81 |
| Stomach | 2.03 ± 0.61 | 1.36 ± 0.60 | 2.51 ± 0.59 | 0.80 ± 0.09 |
| Small Int. | 3.37 ± 0.35 | 1.82 ± 1.20 | 4.55 ± 1.59 | 1.51 ± 0.28 |
| Large Int. | 3.28 ± 0.61 | 1.85 ± 0.58 | 3.41 ± 0.87 | 1.33 ± 0.08 |
| Spleen | 1.09 ± 0.75 | 2.37 ± 0.40 | 0.77 ± 0.10 | 2.07 ± 0.14 |
| Pancreas | 0.49 ± 0.18**^a^** | 0.43 ± 0.12 | 1.19 ± 0.15**^a^** | 0.39 ± 0.03 |
| Brain | 0.08 ± 0.03 | 0.08 ± 0.02 | 0.09 ± 0.01 | 0.05 ± 0.01 |
| Bone | 0.16 ± 0.07 | 0.18 ± 0.10 | 0.11 ± 0.02 | 0.18 ± 0.06 |
| Muscle | 0.26 ± 0.06 | 0.13 ± 0.06 | 0.37 ± 0.07 | 0.12 ± 0.03 |

**Table S1.** Ex vivo tissue distribution of IF-^89^Zr-B12 and ^89^Zr-B12 in mice on a B12 deplete or replete diet at 24 h plotted as %recovered/organ as mean ± SD. a = p ≤ 0.05 between ^89^Zr-B12 replete and deplete mouse models; b = p ≤ 0.05 between IF-^89^Zr-B12 replete and deplete mouse models.

**Figure S5**. Western Results for cubilin in CHO and BN16 cells. Results show cubilin expression in BN16 cells and no expression in CHO cells**.** Lane 1: Thermo Fisher Scientific HiMark Pre-Stained HMW Protein Standard, lane 2: BN16 cell lysate, lane 3: CHO-K1 cell lysate. 1° Ab: Santa Cruz Biotechnology cubilin anti-goat polyclonal (1:200), 2° Ab: Santa Cruz Biotechnology chicken anti-goat HRP conjugated (1:4000).

**Figure S6.** Western blot results for ASGPR in HepG2 cells. Expression was seen in HepG2 cells and not CHO cells. Western Blot 1: CHO-K1 lysate, 2: BioRad Kaleidoscope Protein Markers, 3: HEPG2 Cell lysate was ran on a 12% agarose gel and transferred on a PDVF membrane. Blocked for 1 h and the primary antibody-HRP: 1:200 overnight at 4°C.

**Figure S7.** Flow Cytometry results for uptake of IF-B12-Cy5 and B12-Cy5 in CHO-K1 cells. Neither compound was internalized by CHO-K1 cells indicating no expression of cubilin or CD206. Analysis on a Becton Dickinson LSRII Cell Analyzer. Excitation: 640 nm, Emission: 660/20. Solutions were prepared at 100 nM in HBSS. Red: untreated, Blue: B12-Cy5, Orange: IF-B12-Cy5.

**Figure S8.** Flow Cytometry results for uptake of IF-B12-Cy5 and B12-Cy5 in HepG2 cells. Both compounds were not internalized by HepG2 cells indicating no recognition by any cell receptors. Analysis on a Becton Dickinson LSRII Cell Analyzer. Excitation: 640 nm, Emission: 660/20. Solutions were prepared at 100 nM in HBSS. Red: untreated, Blue: B12-Cy5, Orange: IF-B12-Cy5.


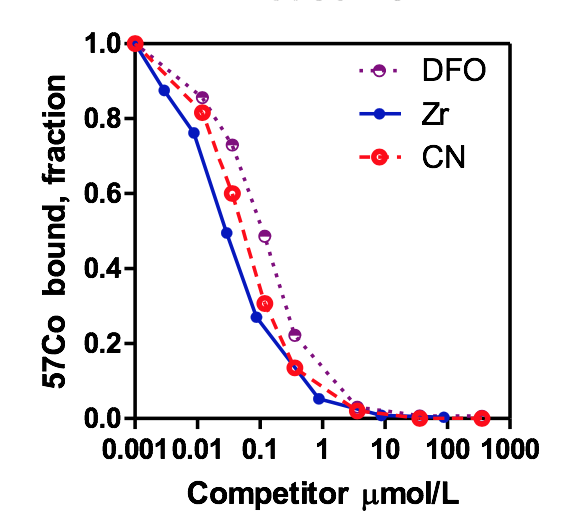


**Figure S9**. Binding affinities of ^91^Zr-DFO-B12 (Zr). B12-DFO (DFO) and CN-B12 (CN) to mouse TC showing comparable binding. Mouse salivary gland extracts was used for source of mouse transcobalamin (TC) (Hygum K *et al*., *PLoS One*. **2011**;6(5):e20638)..
